# Low-Cutoff Frequency Reduction in Neural Amplifiers: Analysis and Implementation in CMOS 65 nm

**DOI:** 10.3389/fnins.2021.667846

**Published:** 2021-06-02

**Authors:** Fereidoon Hashemi Noshahr, Morteza Nabavi, Benoit Gosselin, Mohamad Sawan

**Affiliations:** ^1^Polystim Neurotech. Lab., Department of Electrical Engineering, Polytechnique Montreal, Montreal, QC, Canada; ^2^Department of Computer and Electrical Engineering, Université Laval, Québec, QC, Canada; ^3^School of Engineering, Westlake University, Hangzhou, China; ^4^Institute of Advanced Study, Westlake Institute for Advanced Study, Hangzhou, China

**Keywords:** neural amplifier, low noise, low-power, low-cutoff frequency, compact

## Abstract

Scaling down technology demotes the parameters of AC-coupled neural amplifiers, such as increasing the low-cutoff frequency due to the short-channel effects. To improve the low-cutoff frequency, one solution is to increase the feedback capacitors' value. This solution is not desirable, as the input capacitors have to be increased to maintain the same gain, which increases the area and decreases the input impedance of the neural amplifier. We analytically analyze the small-signal behavior of the neural amplifier and prove that the main reason for the increase of the low-cutoff frequency in advanced CMOS technologies is the reduction of the input resistance of the operational transconductance amplifier (OTA). We also show that the reduction of the input resistance of the OTA is due to the increase in the gate oxide leakage in the input transistors. In this paper, we explore this fact and propose two solutions to reduce the low-cutoff frequency without increasing the value of the feedback capacitor. The first solution is performed by only simulation and is called cross-coupled positive feedback that uses pseudoresistors to provide a negative resistance to increase the input resistance of the OTA. As an advantage, only standard CMOS transistors are used in this method. Simulation results show that a low-cutoff frequency of 1.5 Hz is achieved while the midband gain is 30.4 dB at 1 V. In addition, the power consumption is 0.6 μW. In the second method, we utilize thick-oxide MOS transistors in the input differential pair of the OTA. We designed and fabricated the second method in the 65 nm TSMC CMOS process. Measured results are obtained by *in vitro* recordings on slices of mouse brainstem. The measurement results show that the bandwidth is between 2 Hz and 5.6 kHz. The neural amplifier has 34.3 dB voltage gain in midband and consumes 3.63 μ*W* at 1 V power supply. The measurement results show an input-referred noise of 6.1 μ*V*_*rms*_ and occupy 0.04 *mm*^2^ silicon area.

## 1. Introduction

Neural signal acquisition has a crucial role in understanding the function of the different parts of the brain as well as exploring and treating its various disorders (Stevenson and Kording, 2011). In addition, this data is used in developing the neural prostheses (Sun et al., 2008) and brain machine interfaces (BMI) (Fifer et al., 2012). This is why the demand for new techniques that enable monitoring brain activity wirelessly through implantable devices is increasing every day (Schwartz et al., 2006; Mollazadeh et al., 2009; Cook et al., 2013). A complete review on neural recording is given in Hashemi Noshahr et al. (2020) and Luan et al. (2020).

Brain signals are very small and have very low bandwidth. For instance, the maximum amplitude of local field potentials (LFP) is typically 1 mV and the frequency range is <1 Hz up to 300 Hz (Van Rijn et al., 1991). On the other hand, the amplitude of the spikes or the neural action potentials (AP) are typically as high as 500 μV and their operational frequency is up to 7 kHz (Najafi and Wise, 1986).

Increasing the number of the neural recording sites, which are called channels, is required in some applications, as the spatial resolution of the capturing signals increases. As an example, the total number of channels reported in Musk (2019) is 3072. The electrochemical reaction at the electrode-tissue interface in each channel generates different DC offset voltages across the various electrodes. These voltages vary typically between 1 and 10 mV and in some cases up to 50 mV (Bagheri et al., 2017). As the offset voltages of the channels have high value, they can saturate the neural amplifier. Therefore, they should be eliminated. The most common approach to block this DC input offset is to utilize large AC-coupling capacitors (Harrison and Charles, 2003; Ng and Xu, 2012). On the other hand, there is an alternative method that blocks these DC offset voltages by using a low-pass filter in the feedback path, which is called DC-coupled input offset rejection. The authors in Enz et al. (1995), Yazicioglu et al. (2008), Muller et al. (2012), Biederman et al. (2013), Lee et al. (2019), Jomehei and Sheikhaei (2019), Cabrera et al. (2020), and Farouk et al. (2020) use this method, however, it requires a huge capacitor or high power consumption amplifier in the feedback path.

To design multichannel neural amplifiers, the following factors should be considered and diminished as much as possible.

1. Power consumption: the brain tissues that are surrounded by implantable neuro-amplifiers must be protected from heat damage. For this purpose, the power dissipation of these amplifiers must be lowered.
2. Chip area: The neural amplifiers are generally huge. This is because they usually utilize large AC-coupled input capacitors. Also, to decrease the flicker-noise power of amplifiers, the size of the MOS transistors is designed to be very large especially in the differential pairs. Therefore, for a specific chip area, to maximize the number of the channels, the amplifiers should be designed in their minimum area.
3. Noise: the neural signals have very low amplitude and bandwidth. The flicker and thermal noise of the neural amplifier circuit is the main source of the noise, which can decrease the signal to noise ratio (SNR) in the output of the amplifiers. This is why they are designed as a low noise amplifier (LNA). In the low frequency, the power of the flicker noise is dominant. To decrease the flicker-noise power, in addition to increasing the size of the transistors and utilizing a PMOS differential pair, the chopper-stabilization technique is used (Denison et al., 2007; Verma et al., 2010; Xu et al., 2011; Yazicioglu et al., 2011; Luo et al., 2019; Samiei and Hashemi, 2019). The chopper-stabilization technique modulates the low-frequency noise of the OTA (flicker noise), as well as the offset voltage to a higher frequency by the chopper switches. These higher frequencies are eliminated with a low pass filter (LPF).

The 65 nm CMOS and finer technologies introduce new challenges as a result of the short channel effects for analog circuits. One of these challenges is decreasing the transconductance (gm) of MOS transistors, which diminishes the voltage gain of the whole amplifier. This can be resolved by designing the neural amplifier in 2 or 3 gain stages (Zou et al., 2009; Rezaee-Dehsorkh et al., 2011). The other destructive effect of short channel effects is increasing the low-cutoff frequency (*f*_*L*_) of the AC-coupled neural amplifiers. In this paper, we analyze the parameters that affect the low-cutoff frequency and propose two solutions. The first solution utilizes a standard CMOS and improves the low-cutoff frequency by increasing the input resistance. The second method utilizes thick-oxide transistors to increase the input resistance.

The rest of the paper is organized as follows. Section II analyzes the low-cutoff frequency in neural amplifiers. Section III presents the two proposed solutions. The experimental results are provided in Section IV and the paper concludes in section V.

## 2. Low-Cutoff Frequency Analysis

**Figure 1:**
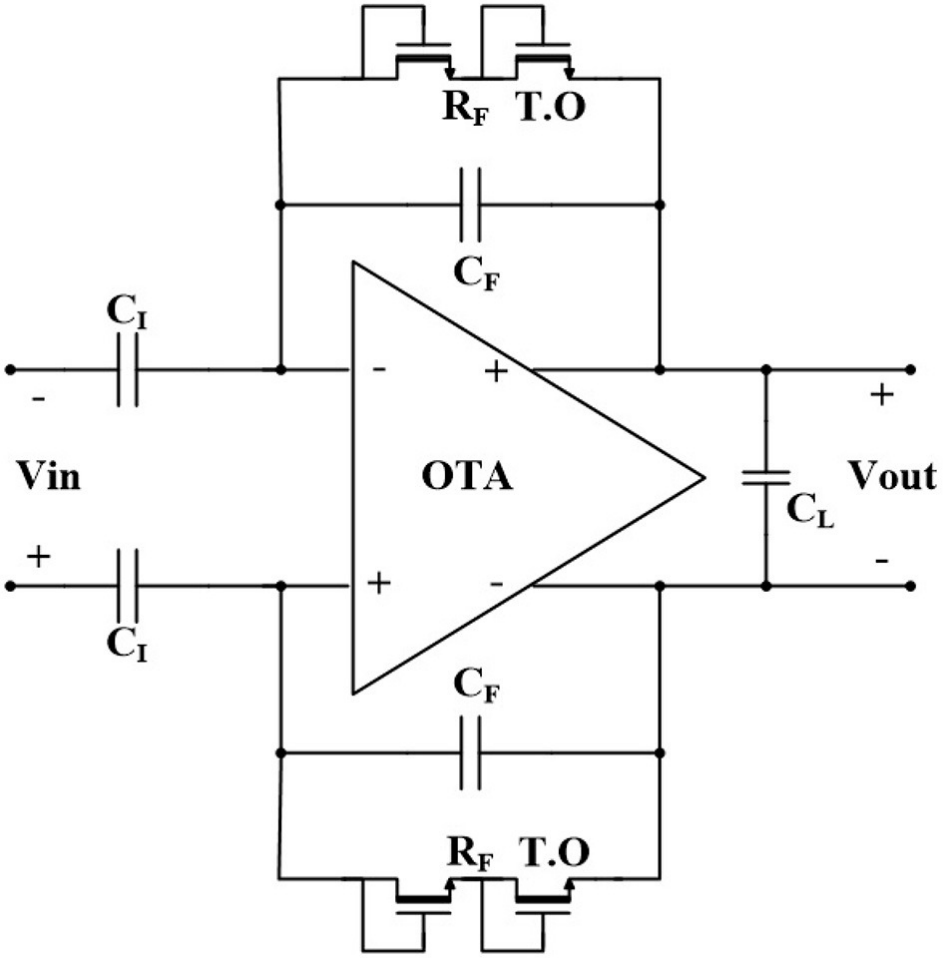
Fully differential capacitive feedback network neural amplifier.

Figure 1 shows the schematic of a fully differential neural amplifier with conventional capacitive feedback network (CFN) architecture. As explained in Harrison and Charles (2003), this architecture is one of the most popular architectures of AC-coupled neural amplifiers in terms of low power consumption, low noise, and compact area. Also, utilizing thick-oxide NMOS pseudoresistors instead of PMOS pseudoresistors, provides a better total harmonic distortion (THD) (Kassiri et al., 2013).

**Figure 2:**
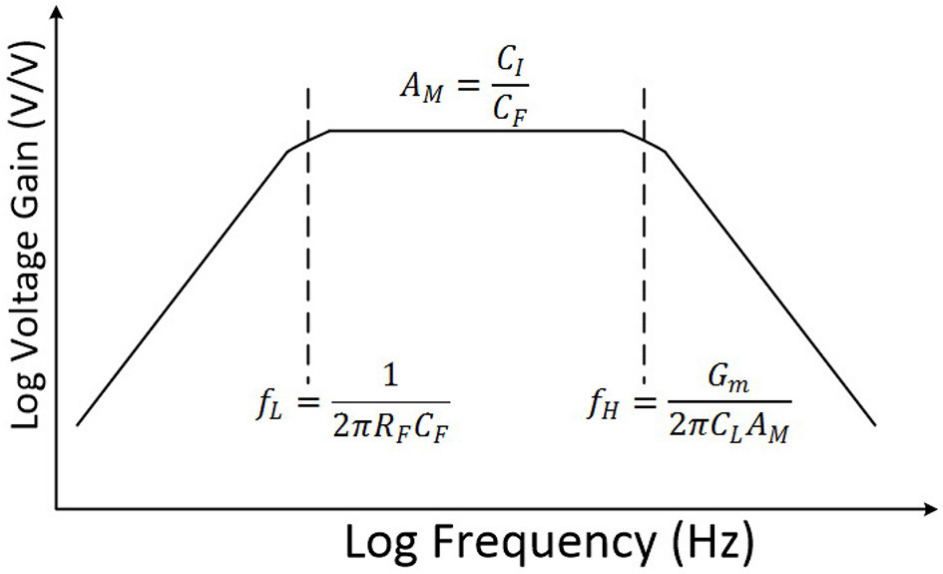
Frequency response of the amplifier.

Figure 2 shows the frequency response of this CFN neural amplifier as a bandpass amplifier. Assuming that the voltage gain of the operational transconductance amplifier (OTA) is significantly high, the voltage gain of the amplifier in the midband (*A*_*M*_) can be approximately calculated by

(1) $$\begin{array}{r} A_{M}=\frac{C_{I}}{C_{F}} \end{array}$$

where *C*_*I*_ and *C*_*F*_ are input and feedback capacitance of the amplifier, respectively. Also, the low-cutoff frequency (*f*_*L*_) of the amplifier can be approximated as

(2) $$\begin{array}{r} f_{L}=\frac{1}{2\pi R_{F}C_{F}} \end{array}$$

where *R*_*F*_ is the dynamic resistance of NMOS pseudoresistors of the amplifier.

As presented in Equation 2, in order to reduce *f*_*L*_, *C*_*F*_ and *R*_*F*_ should be increased. However, by increasing *C*_*F*_, it is required to increase *C*_*I*_ to maintain the same gain which results in huge area loss for each channel of a multi channel device. In addition, this results in the reduction of the input impedance of the neural amplifier.

MOS pseudoresistors can be utilized as a feedback resistance (*R*_*F*_) for their compactness and high resistance. However, the drawback of this technique is that the MOS pseudoresistors provide much less resistance in advanced technology. For example, in an old technology such as 1.5 μm CMOS technology, by utilizing a MOS pseudoresistor for the *R*_*F*_, a *C*_*F*_ of only 200 fF is enough to achieve a *f*_*L*_ of 0.025 Hz (Harrison and Charles, 2003). However, with the same technique and the same value for *C*_*F*_, a *f*_*L*_ of 39 Hz is reported in the 180 nm CMOS technology (Shoaran et al., 2014). Moreover, in the 130 nm CMOS technology (Abdelhalim et al., 2013), a higher *C*_*F*_ of 300 fF is used to compensate for the low *R*_*F*_ to provide a *f*_*L*_ of 0.1 Hz. Moreover, in the 65 nm CMOS technology, our simulation results show that when a *C*_*F*_ of 200 fF is used, the *f*_*L*_ is achieved at 472 Hz. To better understand the effects that increase the *f*_*L*_ value in the advanced CMOS technologies, we provide a small signal analysis of the amplifier in the following.

**Figure 3:**
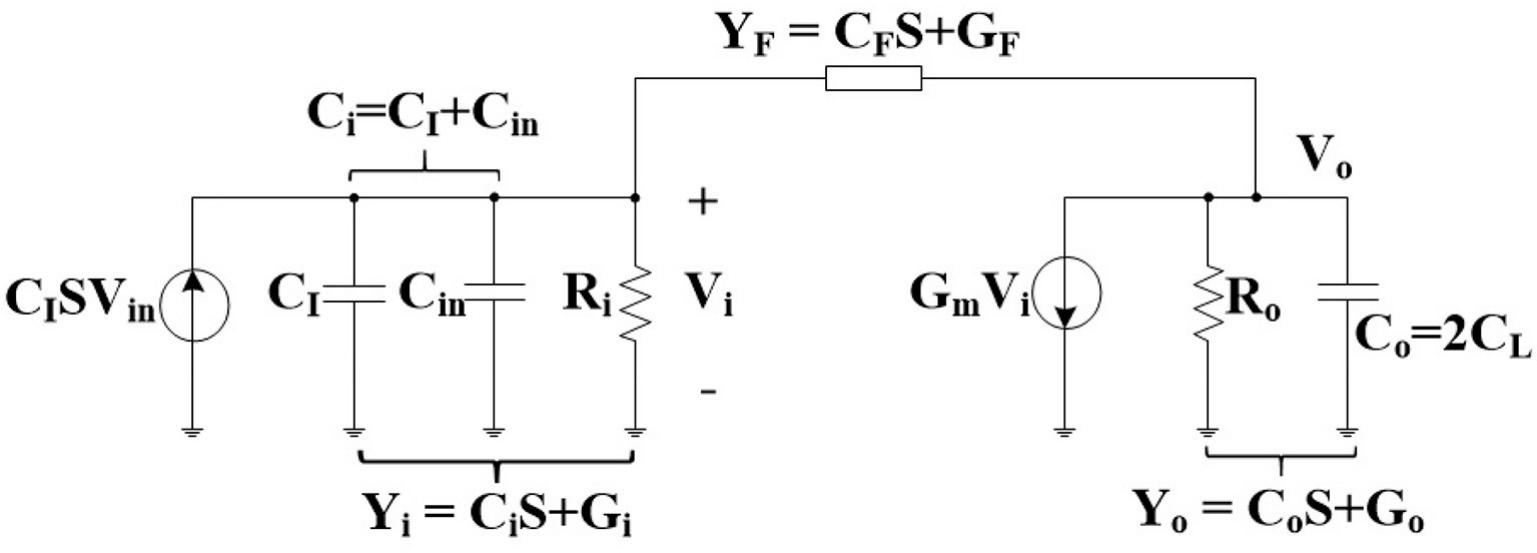
Small signal equivalent of the half-circuit of the neural amplifier.

The equivalent small signal half-circuit of a neural amplifier of Figure 1 is depicted in Figure 3. The OTA can be modeled as a single pole amplifier with a pole at the output node. In this figure, *G*_*m*_ is the transcunductance of the OTA and *C*_*in*_, *R*_*i*_, and *R*_*o*_ are OTA's input terminal capacitance, resistance, and the output terminal resistance, respectively. We extract the time constant of the first pole as

(3) $$\begin{array}{l} \;\tau_{1}=\frac{1}{p_{1}}=\\ =\frac{C_{F}\left(G_{o}+G_{m}\right)+C_{o}G_{F}+C_{i}\left(G_{o}+G_{F}\right)+G_{i}\left(C_{o}+C_{F}\right)}{G_{F}\left(G_{m}+G_{o}\right)+G_{i}\left(G_{F}+G_{o}\right)} \end{array}$$

Reduction of the oxide thickness in advanced technologies translates to lower input resistance (i.e., higher *G*_*i*_) due to higher gate leakage current. By increasing *G*_*i*_, the denominator in Equation (3) grows much faster than the numerator. Therefore, the time constant (τ_1_) increases resulting in lower *f*_*L*_.

However, for older technologies, we can simplify Equations (3) to (4) with the assumption that OTA's input resistance (*R*_*i*_) is infinity (i.e., *G*_*i*_ is approximately zero) (Hashemi Noshahr and Sawan, 2017).

(4) $$\begin{array}{r} \tau_{1}=\frac{1}{p_{1}}=R_{F}C_{F}+\frac{C_{o}R_{o}}{1+G_{m}R_{o}}+\frac{C_{i}\left(R_{F}+R_{o}\right)}{1+G_{m}R_{o}} \end{array}$$

If the gain of the OTA (*G*_*m*_*R*_*o*_) is high, the second and third terms of this equation can be considered negligible resulting in Equation (5) where the corresponding frequency to τ_1_ is the same as Equation (2). In other words, Equation (5) is a special case of Equation (3) where the gain of the OTA is high and the input resistance of the OTA is infinity.

(5) $$\begin{array}{r} \tau_{1}=\frac{1}{p_{1}}=R_{F}C_{F} \end{array}$$

**Figure 4:**
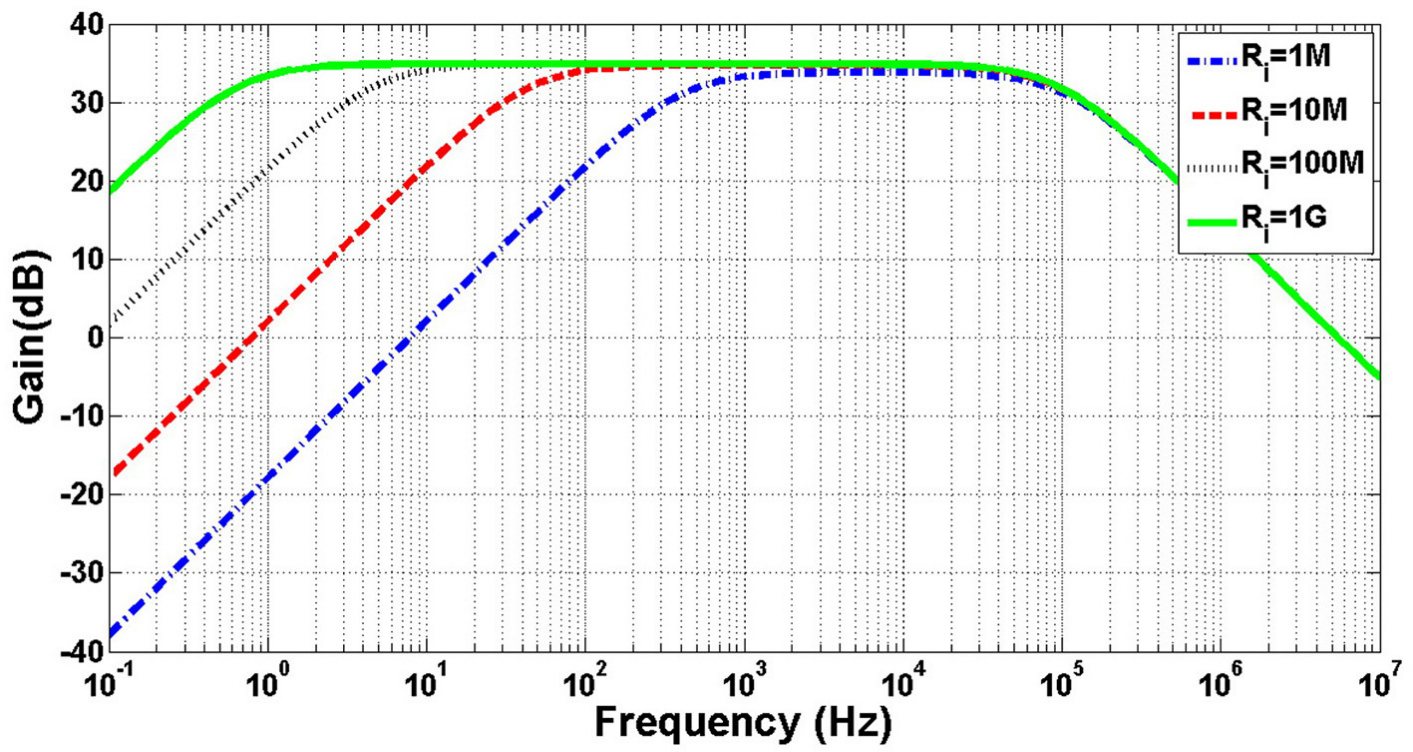
Simulation of frequency response of a neural amplifier with various amounts of *R*_*i*_.

Figure 4 illustrates the frequency response of the small signal model of the amplifier shown in Figure 3 for different values of *R*_*i*_. The DC voltage of the outputs is biased at 0.5 V and thick-oxide NMOS pseudoresistors are utilized for feedback resistors. The values of *G*_*m*_, *R*_*o*_, *C*_*I*_, *C*_*F*_, *C*_*in*_, and *C*_*o*_ are chosen as 22.4 μ℧, 157*MΩ*, 11.5 *pF*, 200 *fF*, 3 *pF*, and 200 *fF*, respectively. As shown in this figure, *f*_*L*_ decreases by increasing *R*_*i*_.

## 3. Proposed Solutions

In this section, we propose two solutions to decrease the low-cutoff frequency down to 1 Hz of OTA's in advanced CMOS technologies without increasing the feedback capacitance (*C*_*F*_).

### 3.1. Cross-Coupled Positive Feedback

**Figure 5:**
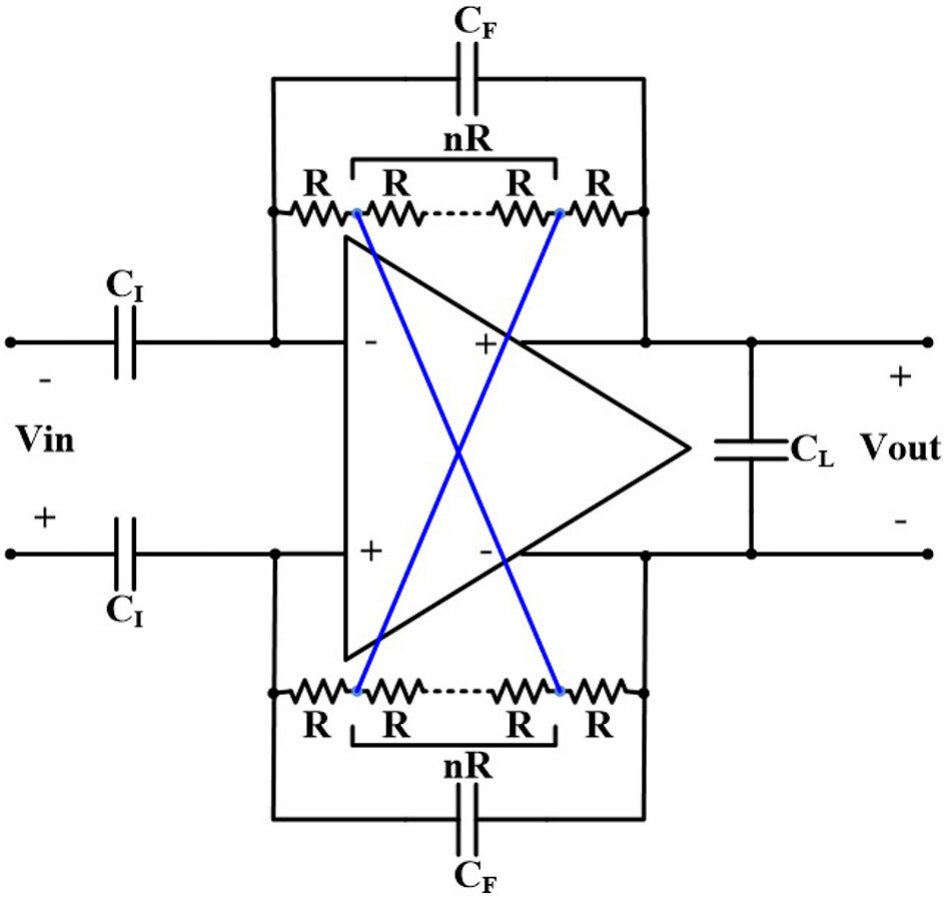
The neural amplifier with cross-coupled positive feedback architecture.

**Figure 6:**
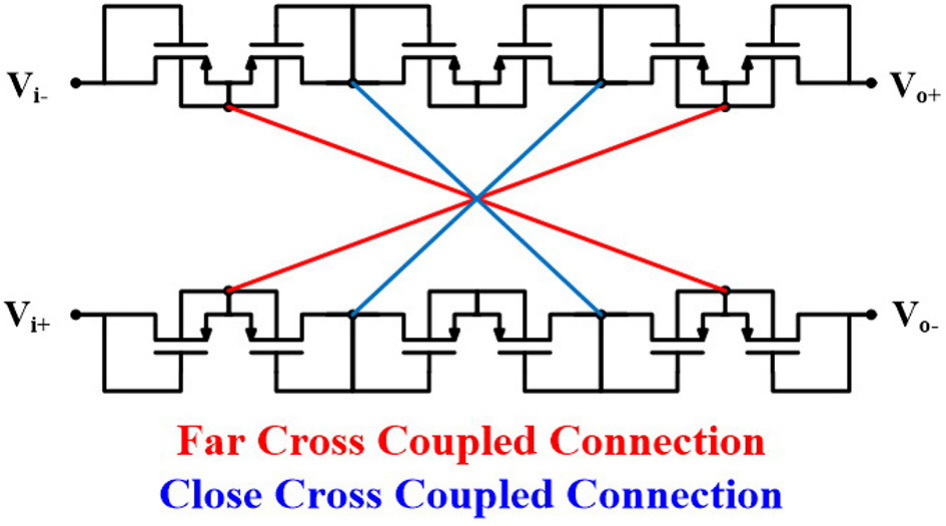
Cross-coupled positive feedback connections.

Figure 5 shows the architecture of the neural amplifier with cross-coupled positive feedback (CCPF) connections in which multiple (n+2) numbers of pseudoresistors are utilized. Figure 6 shows two implementations of the CCPF connections (far and close connections) in which each pseudoresistor is implemented with a standard PMOS transistor. By knowing the fact that the CCPF provides a negative resistance (−|*R*_*N*_|), the equivalent input resistance of the OTA can be presented by

(6) $$\begin{array}{r} R_{ieq}=R_{i}\;||\;\left(-|R_{N}|\right)=\frac{R_{i}|R_{N}|}{|R_{N}|-R_{i}} \end{array}$$

As presented in Equation (6), to maximize *R*_*ieq*_, (|*R*_*N*_| − *R*_*i*_) must be minimized. In other words, to achieve a very high positive equivalent input resistance, the amount of |*R*_*N*_| must be slightly higher than *R*_*i*_, and (|*R*_*N*_| − *R*_*i*_) should approach zero. However, since this negative resistance is created by positive feedback, the stability of the amplifier limits the lower bound of (|*R*_*N*_| − *R*_*i*_).

**Figure 7:**
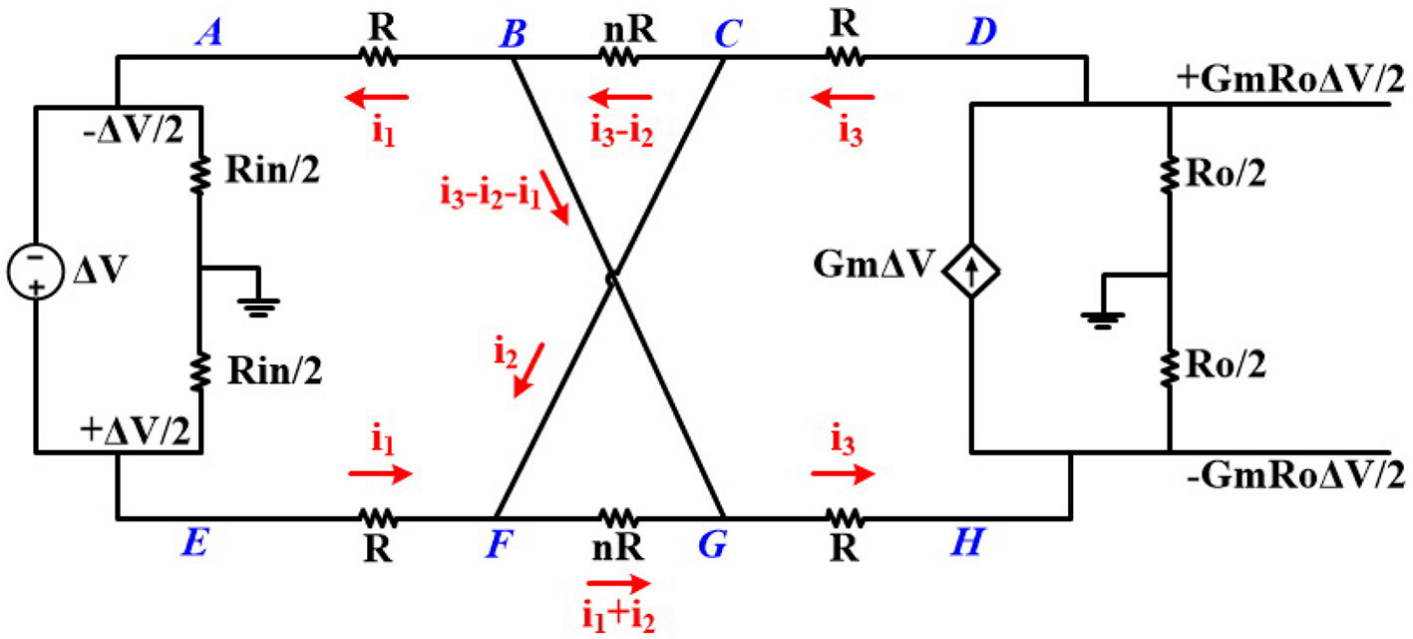
Small signal equivalent circuit of the neural amplifier with a CCPF connection.

To verify Equation (6), we calculate the negative resistance of the CCPF. Figure 7 shows the small signal equivalent circuit of the neural amplifier with a far CCPF connections. For simplicity of calculation, we assume all the pseudoresistors are identical and have the same value.

Performing a KVL in the loops DCBGHD and DCFGHD results in

(7) $$\begin{array}{r} i_{3}=i_{1}+2i_{2} \end{array}$$

Also Performing KVL on the loops of ABCFEA and DCBGHD and considering (Equation 7) results in the following two equations

(8) $$\begin{array}{r} \left(n+2\right)Ri_{1}+nRi_{2}=\Delta V \end{array}$$

(9) $$\begin{array}{r} \left(n+2\right)Ri_{1}+\left(n+4\right)Ri_{2}=G_{m}R_{o}\Delta V \end{array}$$

After solving these equations, the value of *i*_1_ will be

(10) $$\begin{array}{r} i_{1}=\frac{\left(n+4\right)-G_{m}R_{o}n}{4\left(n+2\right)R}\Delta V \end{array}$$

As shown in Figure 7, $R_{N}=\frac{\Delta V}{i_{1}}$ is the equivalent resistance of the whole circuit connected to input terminals of the OTA (nodes A and E), which is parallel to *R*_*in*_. By considering (Equation 10), *R*_*N*_ can be presented as

(11) $$\begin{array}{r} R_{N}=\frac{4\left(n+2\right)R}{\left(n+4\right)-G_{m}R_{o}n} \end{array}$$

By knowing that the gain of the OTA (*G*_*m*_*R*_*o*_) is very high, the dominator of *R*_*N*_ is negative. In practice, the values of the pseudoresistors are not equal and vary based on their currents (or their voltages). Therefore, Equation (11) is not accurate and simulation results are required to calculate the exact value of *R*_*N*_.

**Figure 8:**
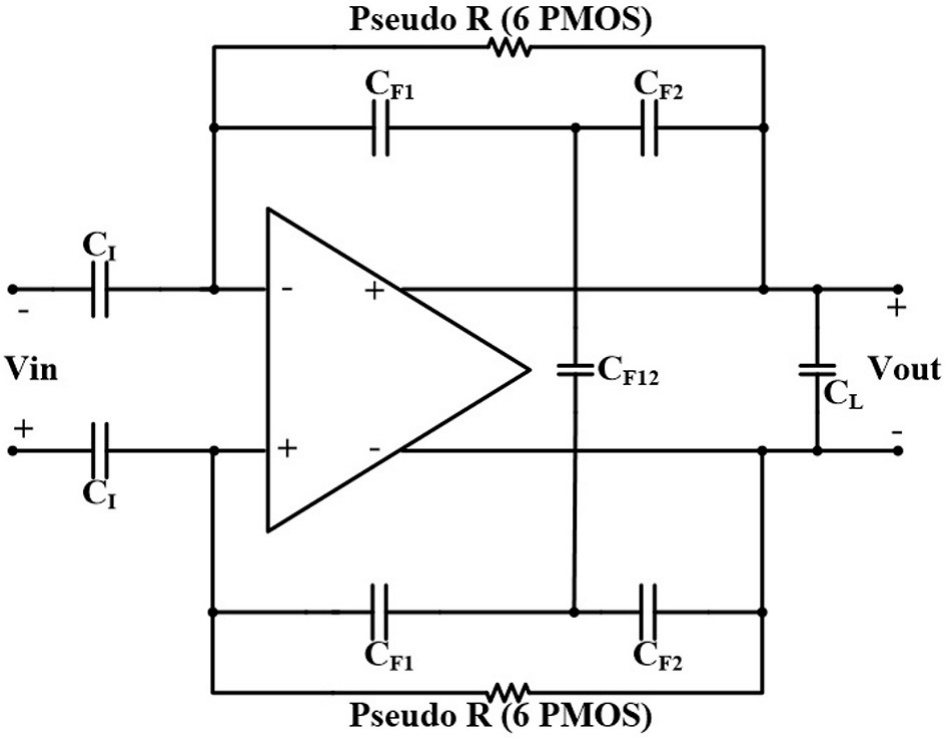
T-capacitor feedback network architecture with CCPF.

The value of the low-cutoff frequency of the amplifier depends on the number and size (W/L) of the pseudoresistors as well as the position of the CCPF connections (far or close). For example, assuming *C*_*I*_ = 10 *pF*, *C*_*F*_ = 200 *fF*, *C*_*L*_ = 1.7 *pF*, and *n* = 4 for a far CCPF connection in the amplifier shown in Figure 5 achieves a *f*_*L*_ of 0.27 Hz with the midband gain of 31.67 dB, while the total capacitance value of this amplifier is 22 pF. In order to decrease the total capacitance, we exploited a T-capacitor feedback network shown in Figure 8 (Ng and Xu, 2013). The pseudoresistors and CCPF connections in this figure are implemented similar to Figure 6 with 6 PMOS transistors.

The midband gain of the amplifier in Figure 8 is calculated as

(12) $$\begin{array}{r} A_{M}=\left(\frac{C_{I}}{C_{F1}}\right)\left(\frac{C_{F1}+C_{F2}+2C_{F12}}{C_{F12}}\right) \end{array}$$

We can adjust the capacitances in Equation (12) to keep the total capacitance of the OTA low while maintaining the same gain. For example, in Figure 8, by choosing the value of the capacitors as *C*_*I*_ = 1.4 *pF*, *C*_*F*1_ = *C*_*F*2_ = 200 *fF*, *C*_*F*12_ = 400 *fF*, and *C*_*L*_ = 200 *fF*, the total capacitor value of the amplifier decreases to 4.2 *pF*, and the low-cutoff frequency increases from 0.27 to 1.5 Hz, which is still in the acceptable range.

**Figure 9:**
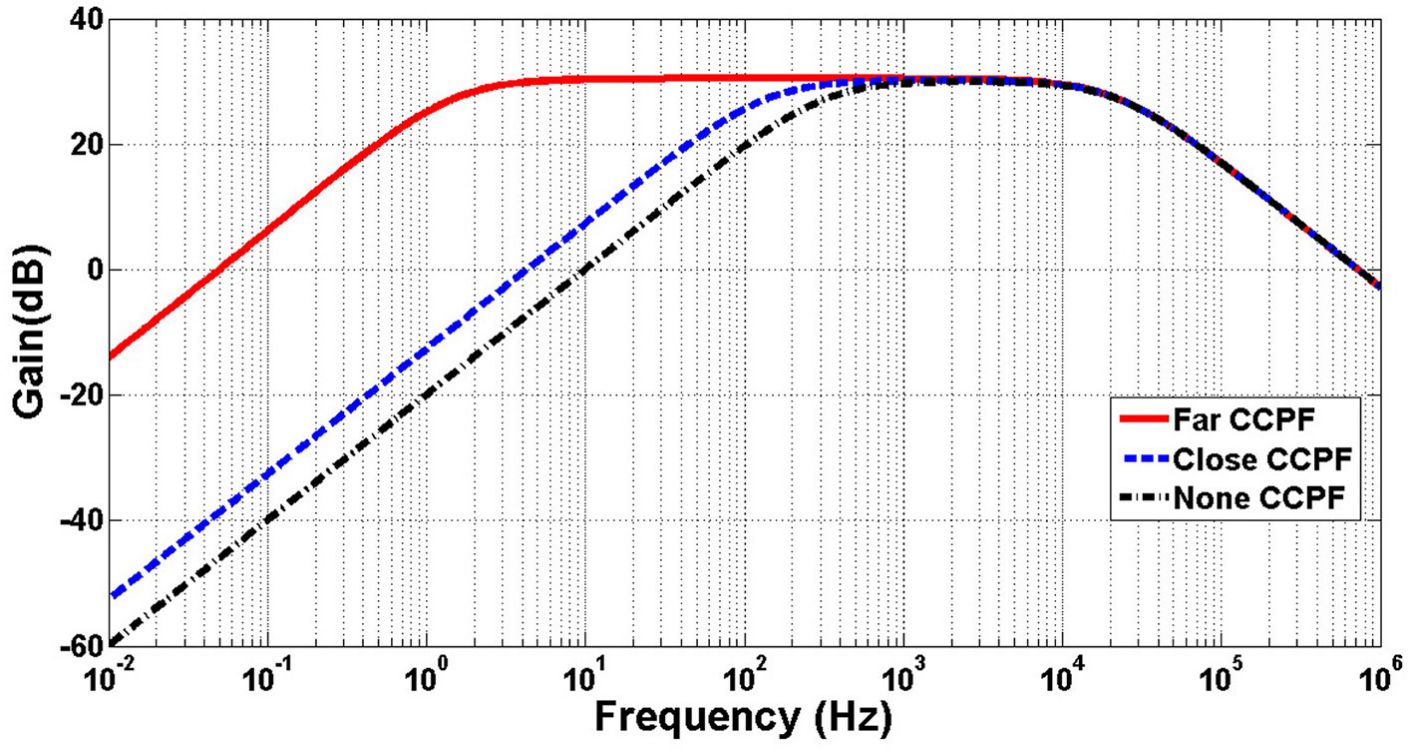
Simulation of frequency response (gain) of the amplifier of Figure 8 with far, close and no CCPF connection.

**Figure 10:**
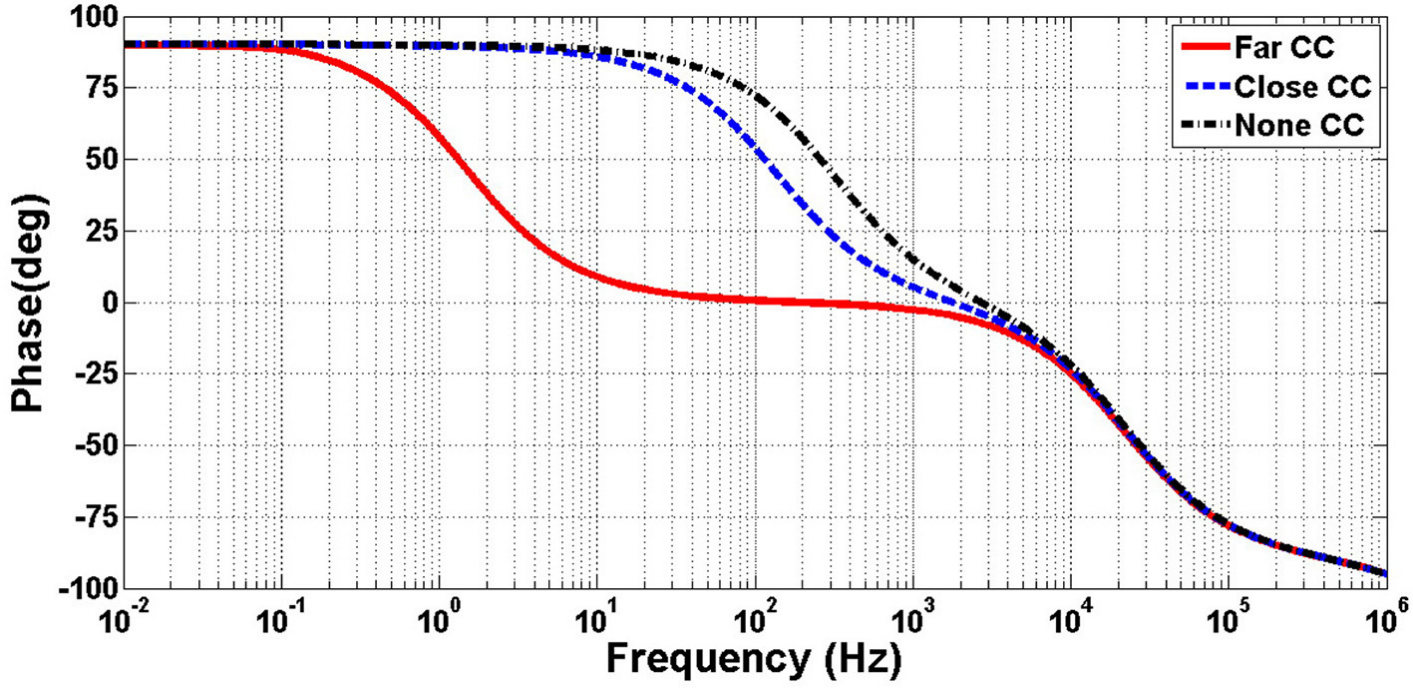
Simulation of frequency response (phase) of the amplifier of Figure 8 with far, close and no CCPF connection.

Figures 9, 10 illustrates the frequency response of the amplifier in terms of gain and phase, respectively, and in in far, close, and no CCPF connections. The amount of the low-cutoff frequency for far, close, and no CCPF connections are 1.5, 143, and 320 Hz, respectively.

**Figure 11:**
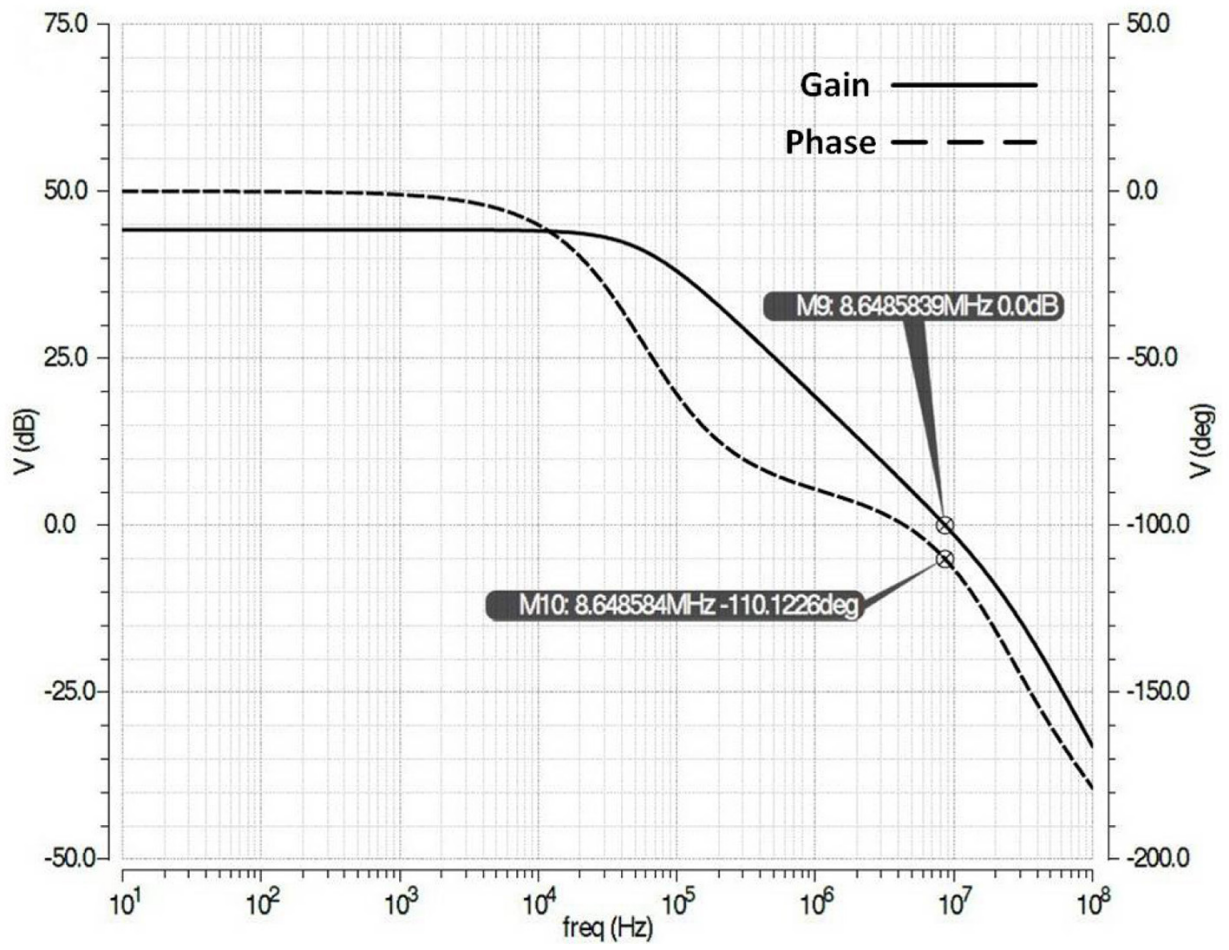
Simulation of open loop frequency response (gain and phase) of the amplifier of Figure 8 with 70 degree phase margin.

The positive feedback in the CCPF architecture of the amplifier can result in instability. However, by carefully designing the number of pseudoresistors, transistor sizes, and the position of the CCPF connection we can make sure that the negative feedback is dominant and the whole architecture is stable and satisfies at least a 60 degree phase margin. Figure 11 shows the simulation of open loop frequency response of the amplifier of Figure 8 with 70 degree phase margin.

By adding switches to the CCPF connection we can program (i.e., turn on or off) the connections in the post-fabrication process. In case of multiple pseudoresistors (e.g., 18), the farther CCPF connections might observe instability due to process variation. Therefore, by programming the connections and choosing closer connections, we can avoid instability. In addition, programmability can also give us control over the value of *f*_*L*_. The closer connections have higher value of *f*_*L*_ and are more stable. On the other hand, the farther connections have lower value of *f*_*L*_ at the cost of less stability.

### 3.2. Thick Oxide Differential Pair

**Figure 12:**
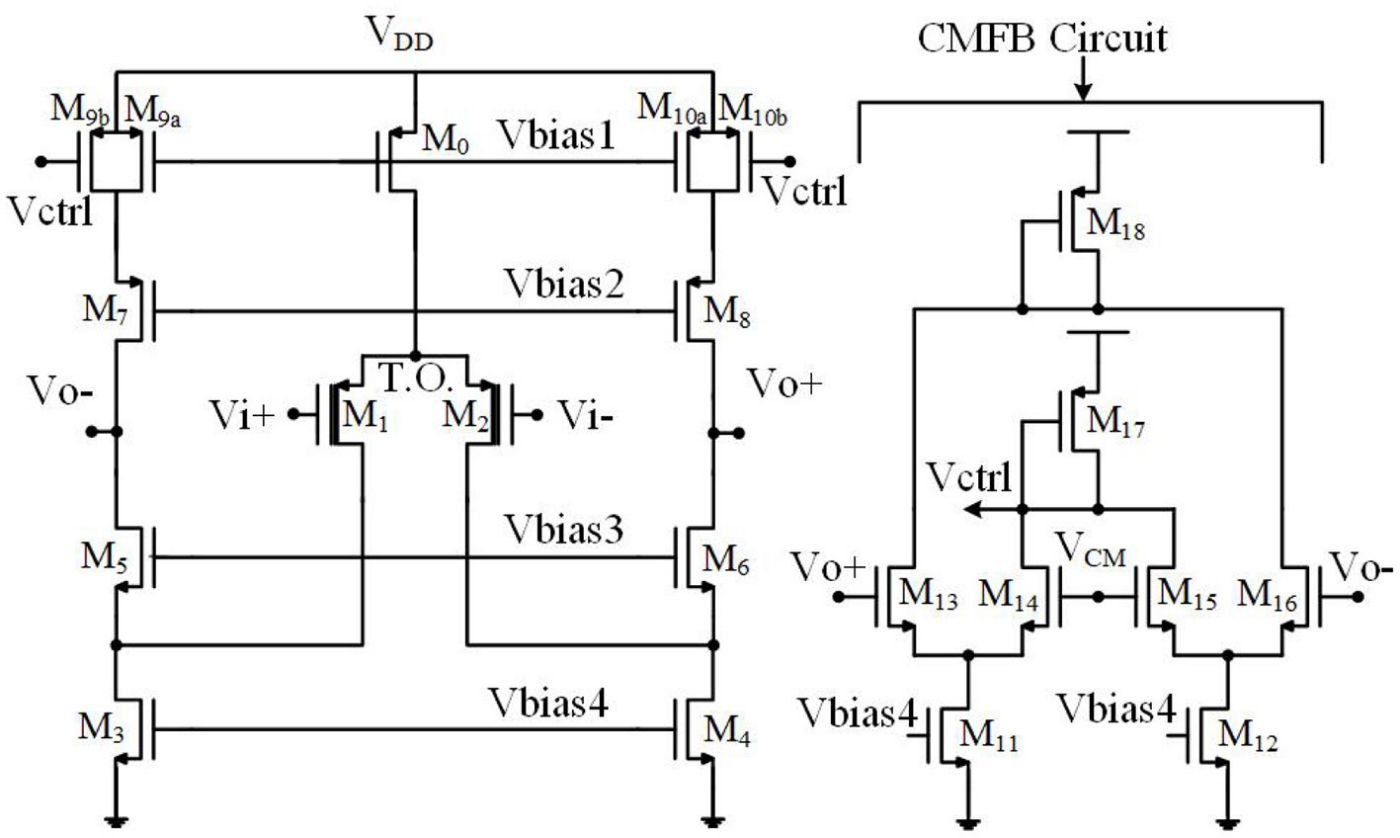
Fully differential folded cascode OTA utilized in neural amplifier.

The second method to increase the input resistance of the OTA without increasing the feedback capacitance is to utilize thick-oxide MOS transistors in the input differential pair. Figure 12 shows the transistor level implementation of the OTA of Figure 1 with thick-oxide PMOS input differential pair. In this figure, the bulks of NMOS transistors are grounded whereas the bulks of PMOS transistors are connected to their sources. The size of each transistor is shown in Table 1 and the bias currents are tabulated in Table 2.

**Table 1:** Transistor sizes of the neural amplifier.

**Transistor**	***W*/*L*(*μm*)**	**Transistor**	***W*/*L*(*μm*)**	**Transistor**	***W*/*L*(*μm*)**
*M*_0_	$5\frac{4}{10}$	*M*_5_	$2\frac{0.8}{2}$	*M*_9*b*_	$4\frac{1}{20}$
*M*_1_	$24\frac{4}{4}$	*M*_6_	$2\frac{0.8}{2}$	*M*_10*a*_	$4\frac{1}{20}$
*M*_2_	$24\frac{4}{4}$	*M*_7_	$2\frac{0.8}{2}$	*M*_10*b*_	$4\frac{1}{20}$
*M*_3_	$3\frac{1}{20}$	*M*_8_	$2\frac{0.8}{2}$	*M*_11_	$2\frac{0.5}{20}$
*M*_4_	$3\frac{1}{20}$	*M*_9*a*_	$4\frac{1}{20}$	*M*_12_	$2\frac{0.5}{20}$
*M*_13_	$1\frac{1}{1}$	*M*_14_	$1\frac{1}{1}$	*M*_15_	$1\frac{1}{1}$
*M*_16_	$1\frac{1}{1}$	*M*_17_	$4\frac{1}{20}$	*M*_18_	$4\frac{1}{20}$

**Table 2:** Bias currents of the neural amplifier.

***I*_*M*0_**	***I*_*M*3, 4_**	***I*_*M*9*a*, 10*a*_**	***I*_*M*11, 12_**
1.83μA	1.276μA	175nA	390nA

**Figure 13:**
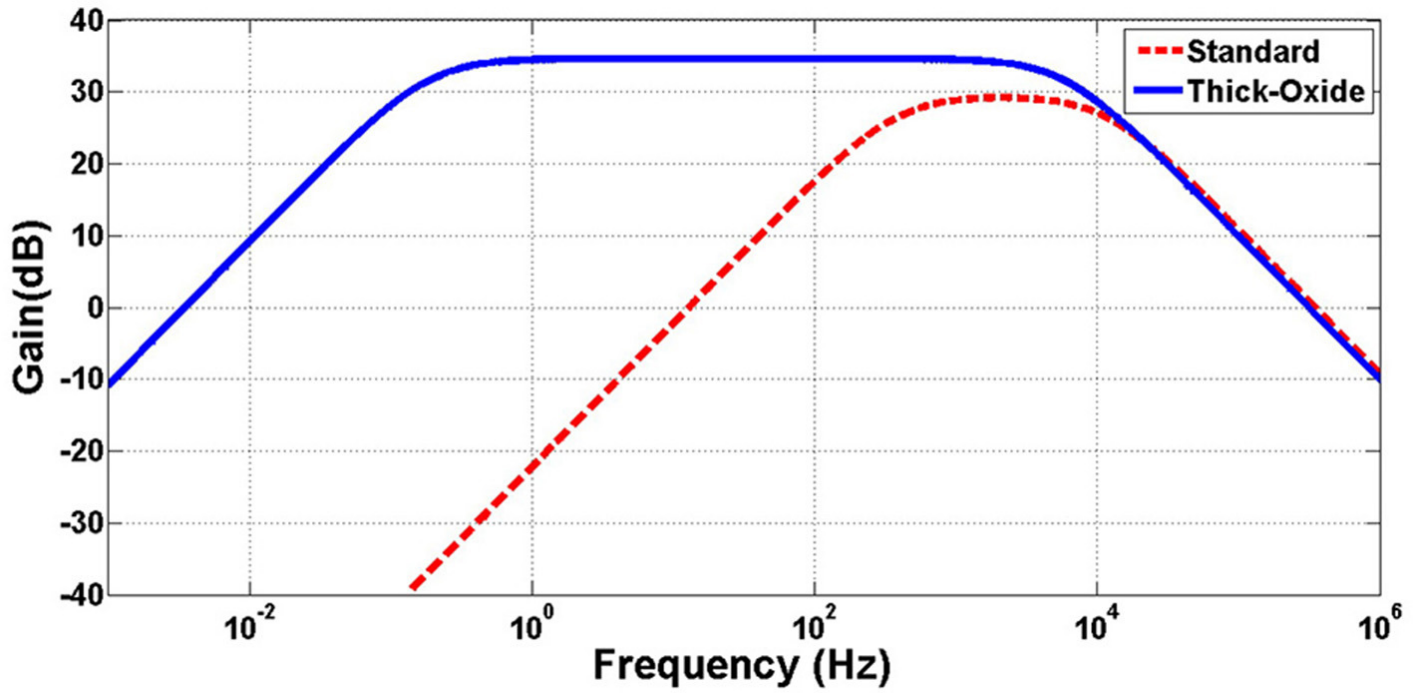
Simulation of frequency response of neural amplifier with thick-oxide and standard PMOS differential pair.

Figure 13 shows the simulation results of the designed neural amplifier utilizing the OTA of Figure 12 and the OTA with standard PMOS input differential pair. The gain of the OTA and the whole neural amplifier are 68.2 and 34.6 dB, respectively. As shown in this figure, applying a thick-oxide PMOS in the input differential pair improved the low-cutoff frequency from 360 to 0.19 Hz. These simulation results confirm that increasing the input resistance of the OTA by utilizing thick-oxide PMOS in the differential pair decreases the low-cutoff frequency dramatically.

In order to increase the SNR of the neural amplifier, the first stage of a neural amplifier is designed as an LNA. To reduce the flicker noise of the OTA of Figure 12, we optimize the size of the PMOS transistors in the input differential pair (i.e., *M*_1_ and *M*_2_). Also, as mentioned in Harrison and Charles (2003), to minimize the thermal noise, the transistors *M*_1_ and *M*_2_ are biased in the sub-threshold region to maximize their transconductance over drain current called transconductance efficiency (*g*_*m*_/*I*_*D*_), and the transistors *M*_3_, *M*_4_, *M*_9*a*_, *M*_9*b*_, *M*_10*a*_, and *M*_1_0__*b*__ are biased in the saturation region to minimize their *g*_*m*_/*I*_*D*_.

As mentioned earlier, the bandwidth and operating frequency of neural amplifiers are very low, therefore the dominant noise power is the flicker noise. Also, in the OTA of Figure 12, the differential pair transistors are the main source of the flicker noise in comparison with other transistors (Razavi, 2005). Therefore, to analyze the noise of the proposed neural amplifier, we only investigate the effect of the thick-oxide PMOS differential pair. Utilizing thick-oxide PMOS transistors in the differential pair of the OTA decreases the gate-oxide capacitance per unit area (*C*_*ox*_) due to the increased gate oxide thickness (*t*_*ox*_). Utilizing the thick-oxide PMOS in the input differential pair increases the flicker noise power due to decreasing *C*_*ox*_. The relation between the input-referred noise of the whole neural amplifier ($\bar{V_{ni,amp}^{2}}$) and the OTA input-referred noise ($\bar{V_{ni}^{2}}$) is presented as

(13) $$\begin{array}{r} \bar{V_{ni,amp}^{2}}={\left(\frac{C_{I}+C_{F}+C_{in}}{C_{I}}\right)}^{2}.\bar{V_{ni}^{2}} \end{array}$$

Decreasing the *C*_*ox*_ due to utilizing the thick-oxide PMOS differential pair, increases $\bar{V_{ni}^{2}}$ and decreases the *C*_*in*_ in Equation (13). Since the increase in $\bar{V_{ni}^{2}}$ is much higher than the reduction of its coefficient, the $\bar{V_{ni,amp}^{2}}$ increases by decreasing the *C*_*ox*_. To compensate this drawback, we can increase the gain of the LNA (*C*_*I*_/*C*_*F*_) by increasing *C*_*I*_ to reduce the $\bar{V_{ni,amp}^{2}}$ in Equation (13). Simulation results show that the minimum input-referred noise voltage of the neural amplifier is 5.9 μ*V*_*rms*_ in the frequency range between 1 Hz and 5.6 kHz (bandwidth).

Note that to further reduce the noise of the OTA, it is required to apply noise reduction techniques such as the chopper stabilization technique, which is out of the scope of this paper.

**Figure 14:**
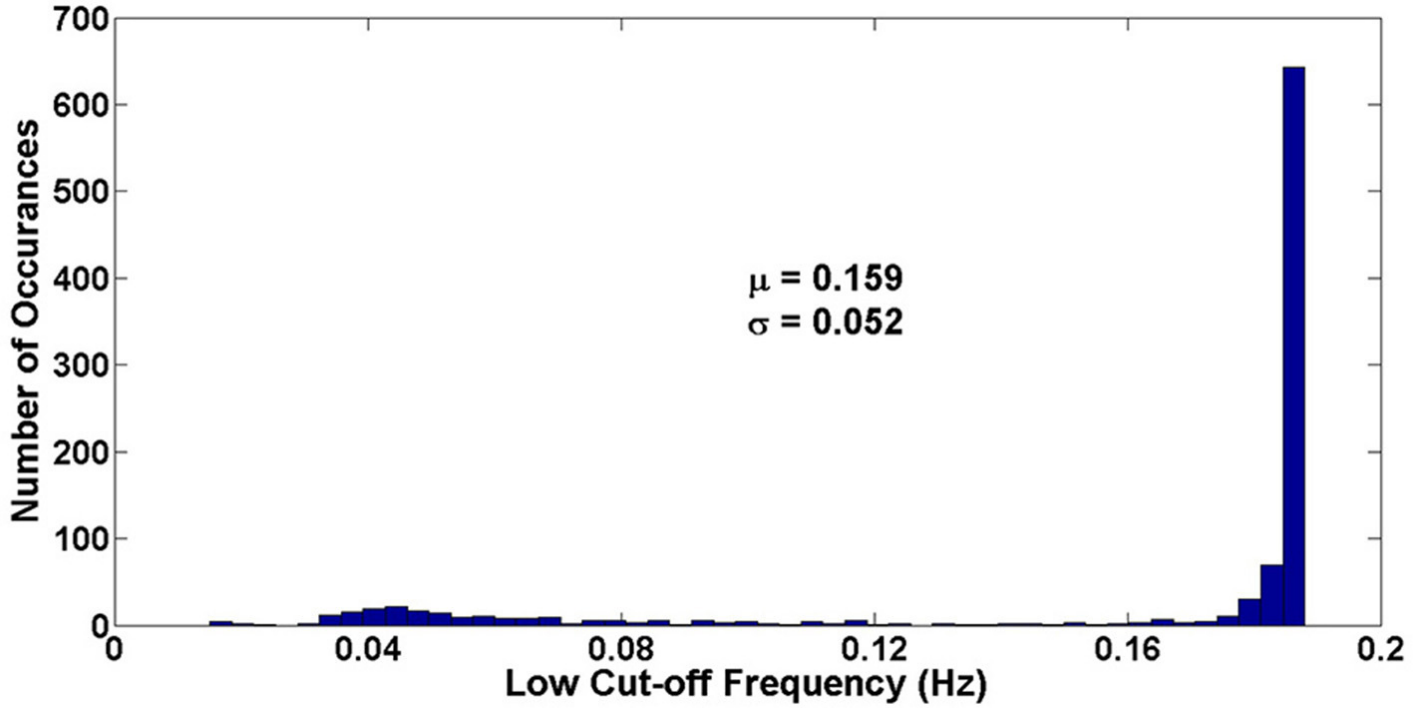
Monte Carlo simulation of low-cutoff frequency of the neural amplifier.

Figure 14 shows the Monte Carlo simulation results (*N* = 1,000) of the low-cutoff frequency. As shown in this figure, the μ is equal to 0.159 Hz and the σ is equal to 0.052, resulting $\frac{3\sigma }{\mu }$ of 0.983.

**Figure 15:**
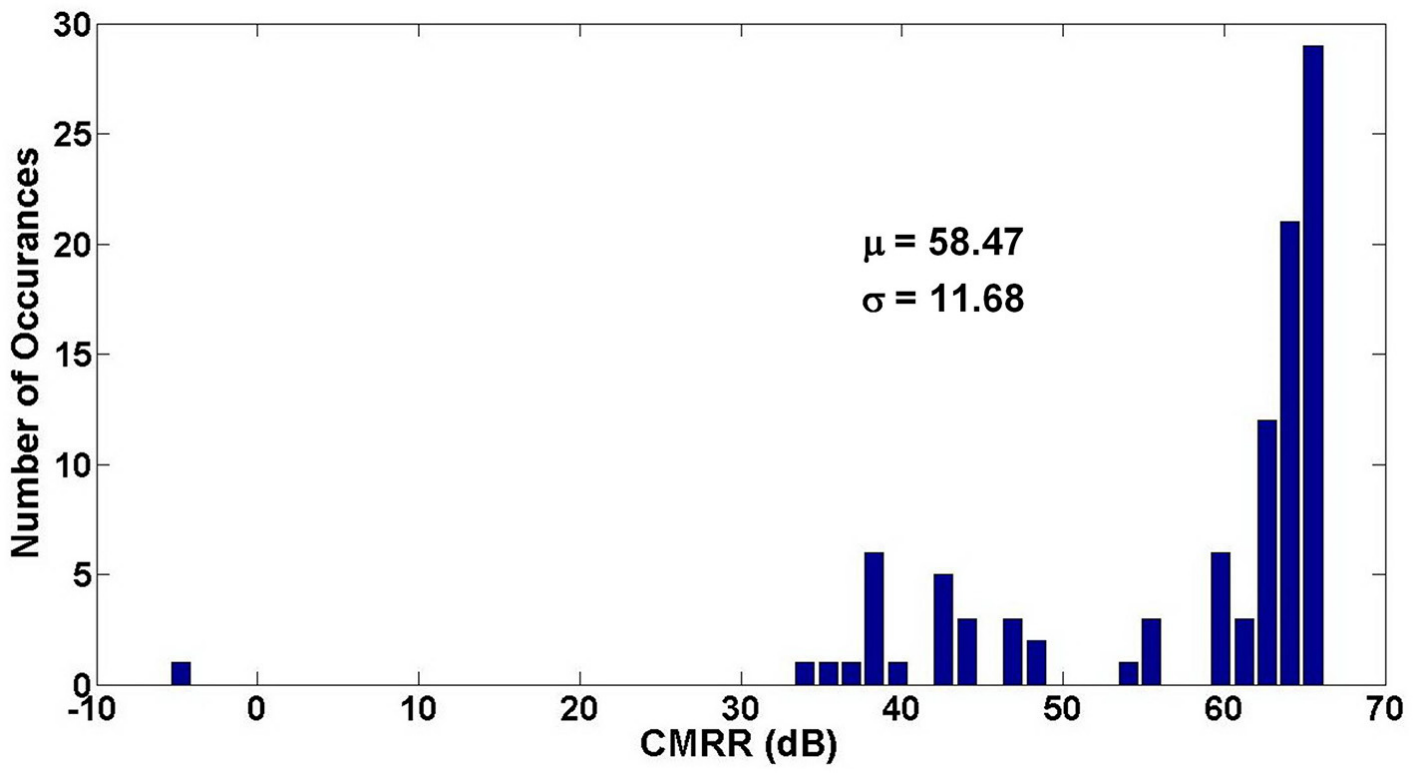
Monte Carlo analysis of CMRR of the neural amplifier.

**Figure 16:**
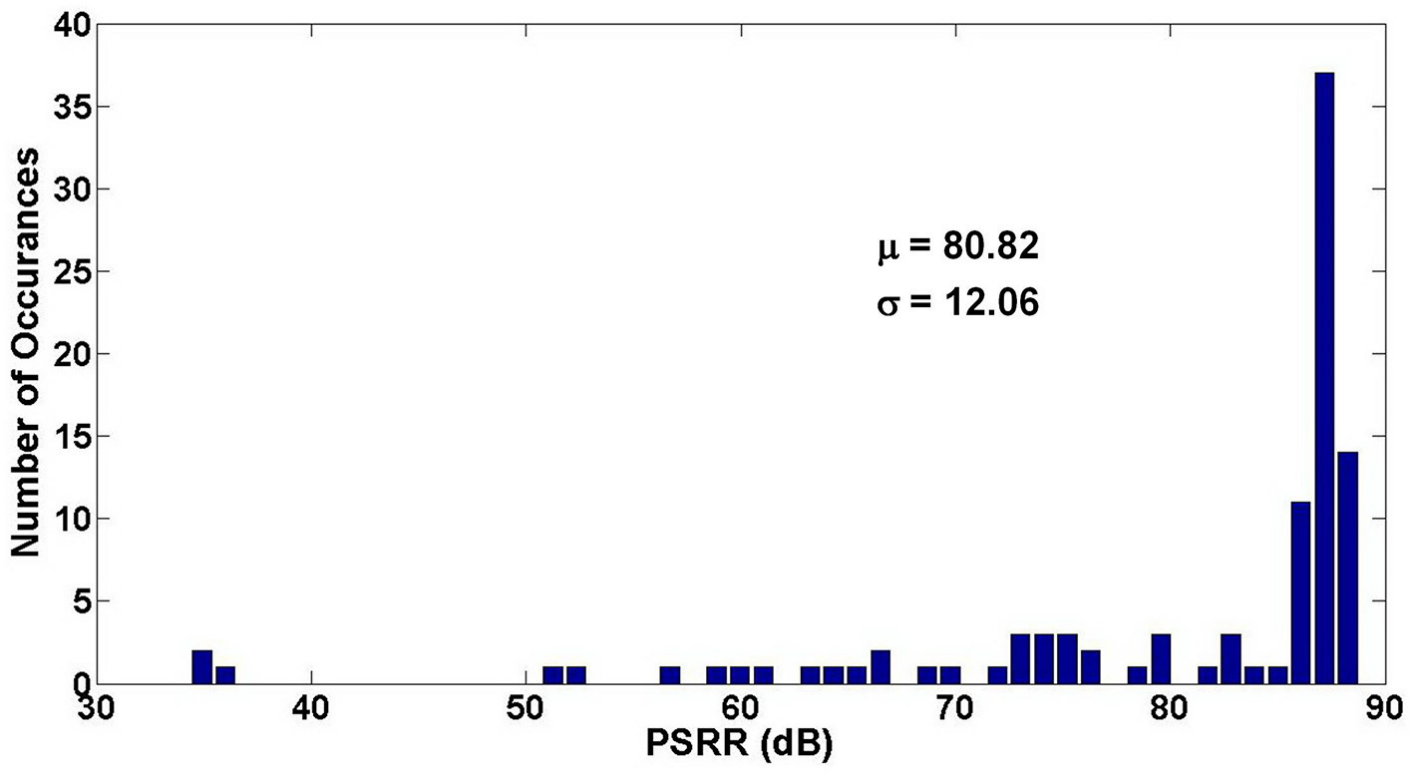
Monte Carlo analysis of PSRR of the neural amplifier.

Figures 15, 16 show the Monte Carlo analysis of CMRR and PSRR of the Neural amplifier. Applying thick-oxide MOS transistors in the input differential pair decreases the gate leakage current significantly and increases the input impedance of the OTA and consequently the CMRR and PSRR improve.

## 4. Measurement and *in vitro* Results

### 4.1. Measured Performance

**Figure 17:**
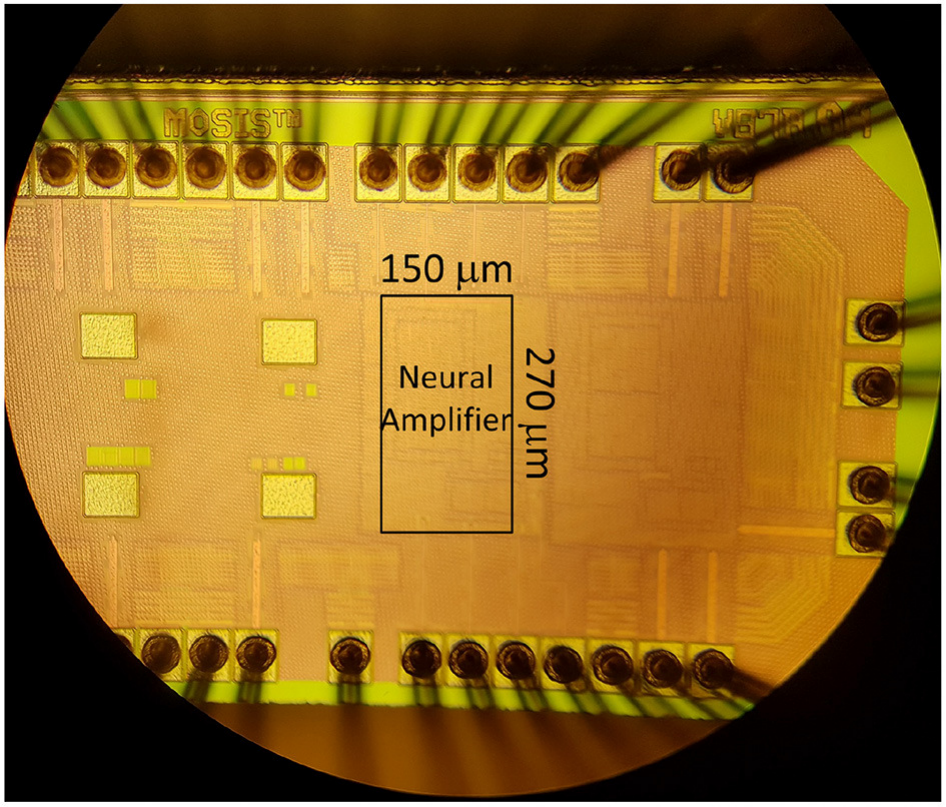
Micrograph of chip containing the neural amplifier with 270 μm × 150 μm die area.

The prototype is implemented in the TSMC 65 nm CMOS process. The *C*_*I*_ and *C*_*F*_ are set to 11.5 pF and 208 fF, respectively, in the layout to achieve a gain of 55 V/V (or 34.3 dB) $\left(A_{M}=\frac{C_{I}}{C_{F}}\right)$. The prototype uses 0.04 *mm*^2^ (270 μm × 150 μm) of silicon area. The micrograph of the die containing the amplifier is shown in Figure 17.

**Figure 18:**
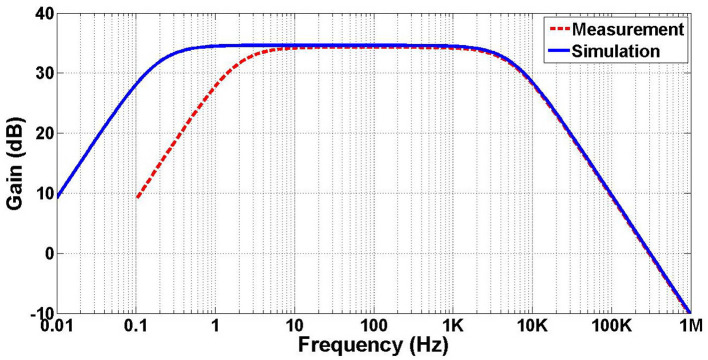
Measured and simulated frequency response of the amplifier. The measured midband gain is 34.3 dB, and the low and high-cutoff frequencies occur at 2 Hz and 5.6 kHz, respectively.

The measured frequency response from 0.1 Hz to 1 MHz is performed through saline medium to mimic the brain environment as well as the simulation result are illustrated in Figure 18. The midband gain is 34.3 dB and the low and high-cutoff frequencies are 2 Hz and 5.6 kHz, respectively. The simulated low-cutoff frequency is 0.19 Hz which is less than that achieved in the measurement result. This deviation is expected as the MOS pseudoresistors are nonlinear and significantly sensitive to their operating point (Harrison and Charles, 2003).

**Figure 19:**
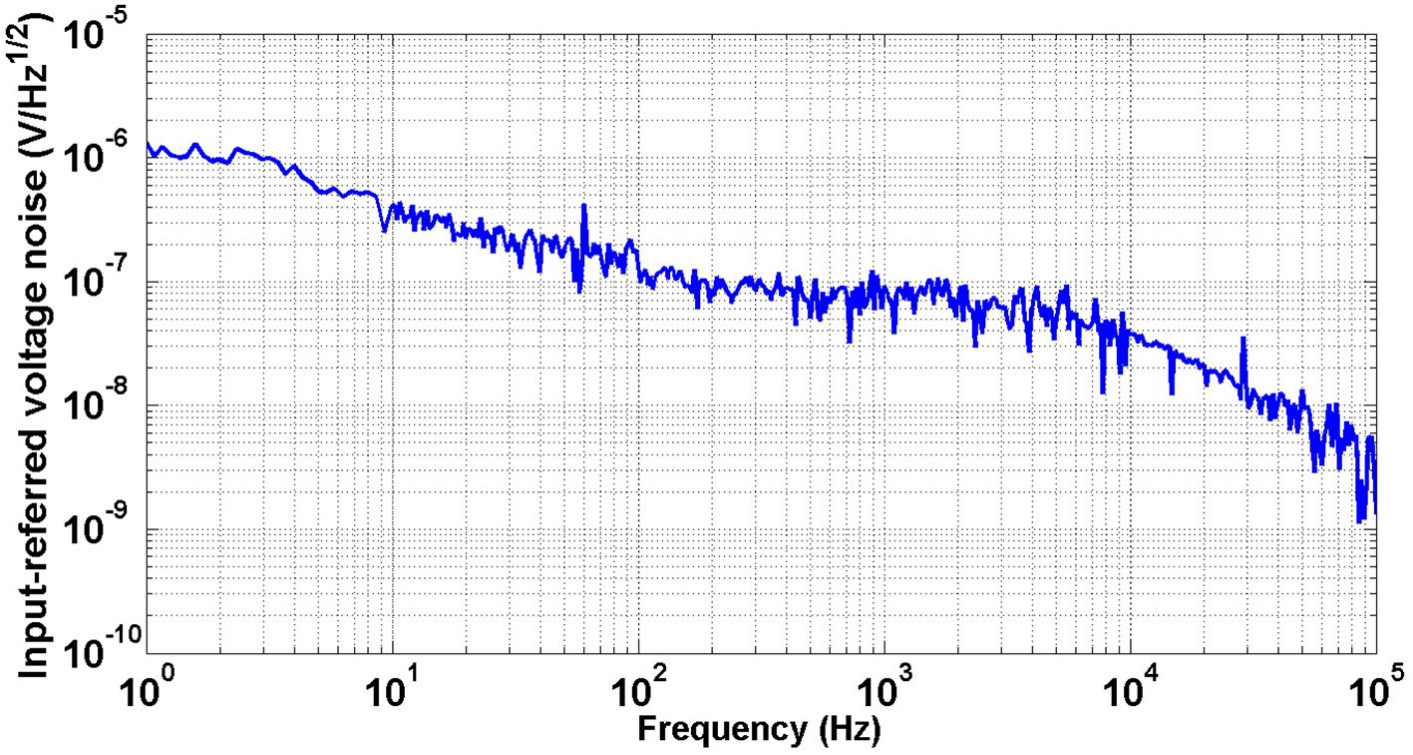
Measured input-referred noise voltage spectrum.

Figure 19 shows the measured input-referred noise voltage spectral density of the neural amplifier. The RMS value of the input referred noise is achieved as 6.1 μ*V*_*rms*_ by integrating the area under the curve from 1 Hz to 5.6 kHz (amplifier bandwidth) in Figure 19. This value is slightly higher than the simulated result (5.9 μ*V*_*rms*_).

Table 3 shows a summary of the simulated and measured parameters of the prototype. A comparison of our work and the other published works is presented in Table 4. All of the chosen neural amplifiers are AC-coupled. To fairly compare these amplifiers with different gain values, number of stages and technology, we only consider the first stage of each amplifiers.

**Table 3:** Experimental and simulation characteristics of neural amplifier.

**Parameter**	**Simulation**	**Measured**
Supply voltage [V]	1	1
Supply current [μ*A*]	3.63	3.63
Gain [dB]	34.6	34.3
Band width [kHz]	5.8	5.6
Low-cutoff frequency [Hz]	0.19	2
Input-Referred Noise [μ*V*_*rms*_]	5.9	6.1
Noise efficiency factor	5.8	6.1
THD (2 *mV*_*pp*_ at 1 kHz) [%]	0.18	<1

**Table 4:** Comparison of fully integrated neural amplifiers.

**Parameter**	**Song et al. (2013)**	**Abdelhalim et al. (2013)**	**Xiao et al. (2010)**	**Biederman et al. (2015)**	**Ng and Xu (2016)**	**Kim and Ko (2019)**	**This work**
Technology [CMOS]	0.18 μm	0.13 μm	0.13 μm	65 nm	65 nm	0.18 μm	65 nm
Area [*mm*^2^]	0.16	N/A	0.4^*^	N/A	0.042	N/A	0.04
Supply [V]	1.2	1.2	0.8	1	1	0.6	1
Power consumption [μ*W*]	0.43	4.5	0.64	1.2	3.28	0.27	3.63
Gain [dB]	26	31.8	49 ^**^	26	26.4	14-28^***^	34.3
BW [Hz]	80-15k	0.1-5k	100^*^ - 6.2k	10-8k	1-8.2k	6.4-4.46k	2-5.6k
*C*_*F*_ [fF]	N/A	300	N/A	500	350	100	208
Input-referred noise [μ*V*_*rms*_]	8.1	6.5	14	7.5	4.13	10.68	6.1
Noise BW [Hz]	80-15k	10-5k	100^*^ - 6.2k	100-10k	1-8.2k	1-10k	1-5.6k
NEF	1.52	7.2	6.5	3.6	3.19	1.79^***^	6.1
CMRR [dB]	>60	75	59	N/A	>90 @100Hz	61.3	66.3^****^
PSRR [dB]	>80	N/A	71	N/A	78 @1kHz	77.2	88^****^
THD	0.05% @10 *mV*_*pp*_	-51dB @1KHz 0.7 *V*_*opp*_	@1*mV*_*pp*_ <0.4%	N/A	1% 0.7*mV*_*p*_	0.5% @ 200 *mV*_*opp*_	<1% @1KHz 2*mV*_*pp*_

* *Estimated*.

** *This gain is reported for two stages. All other gains are reported for the first stage*.

*** *Single CCIA*.

**** *Simulation result*.

Measurement results show that the achieved gain is the highest among all in Table 4. Note that the gain for Xiao et al. (2010) is reported for two stages. Also, the area of the fabricated chip is less than others. However, we should note that comparing the chip area itself without considering the midband gain is not a fair comparison. The midband gain (*A*_*m*_) of the amplifier is equal to $\frac{C_{I}}{C_{F}}$. The low-cutoff frequency (*f*_*L*_) is determined by *C*_*F*_, and *C*_*I*_ is determined by the gain and *C*_*F*_. Also, note that the main contributor to the chip area is *C*_*I*_. In other words, for a normalized gain, lower *C*_*F*_ results in less chip area. Therefore, comparing *C*_*F*_ is a better figure of merit for comparing the chip area while the amplifiers have different gains. In this case, the values of *C*_*F*_ of the proposed amplifier and Ng and Xu (2016) are 208 fF and 350 fF, respectively. Note that the gain reported in our work is 34.3 dB, while the gain in Ng and Xu (2016) is 26.4 dB. This is why the total area of our work is almost the same as that of Ng and Xu (2016).

The amplifier of Song et al. (2013) has been implemented in the 0.18 μm technology with a gain of 26 dB. Its high pass pole is 80 Hz. The value of *C*_*F*_ is not reported, however, the total area of the amplifier is 0.16 *mm*^2^ which is significantly large. In Abdelhalim et al. (2013), neural amplifiers with a gain of 54–60 dB in two gain stages have been implemented in the 0.13 μ*m* process. The first stage (LNA) with the estimated gain of 31.8 dB has 300 fF feedback capacitors with 0.1 Hz low-cutoff frequency. Our analysis shows that the *C*_*F*_ in Abdelhalim et al. (2013) could be reduced to 200 fF if the thick-oxide differential pair is used.

The neural amplifier of Xiao et al. (2010) has employed two gain stages to obtain 49 dB in the 0.13 μ*m* process. The value of the *C*_*F*_ is not reported. However, the estimated amplifier area and *f*_*L*_ are 0.4 *mm*^2^ and 100 Hz, respectively. This amplifier occupies a very large area and has a high low-cutoff frequency. The designs in Biederman et al. (2015) utilize LNA with a gain of 26 dB fabricated in the 65 nm CMOS Technology. It employs a 500 fF feedback capacitor parallel to a pseudoresistor in a conventional CFN architecture similar to our work. The low-cutoff frequency *f*_*L*_ is adjustable, with the minimum value of 10 Hz. The neural amplifier consists of a variable gain amplifier (VGA) and buffer to achieve a gain of 45–60 dB. The amplifier in Ng and Xu (2016) has been implemented with two gain stages with 52.1 dB midband gain in the 65 nm technology. The gain in the first stage, LNA, is 26.4 dB and the *f*_*L*_ is reported as 1 Hz. The LNA exploits a CMOS-inverter-based OTA with 360 fF as *C*_*F*_. The amplifier designed in Kim and Ko (2019) utilizes relatively small transistors in the OTA. In addition to small transistors, an older process of 0.18 μ*m* is used which they both help decreasing the gate leakage and increase the input resistance of the OTA. This results in a low *f*_*L*_ of 6.4 Hz. However, this comes at the cost of high input-referred noise voltage (10.68 μ*V*_*rms*_). CMRR and PSRR in the typical corner simulation are 66.3 and 88 dB, respectively. As mentioned earlier, when thick-oxide CMOS is used, the CMRR and PSRR increase compared to the case when standard CMOS is used. This increase is due to the increased input impedance of the OTA. Also, because of less short channel effects in thick-oxide MOS transistors, the linearity and THD of the amplifier are improved.

### 4.2. *In vitro* Neural Recording

We used this neural amplifier for neural recordings in an *in vitro* experiment on the slices of a mouse brain at the faculty of Dentistry at University of Montreal. A micropipette is used to capture the electrical activity of the brain. The micropipette is filled with NaCl (0.5 mol) without bubbles. This micropipette contains a metal electrode of AgCl which records the extracellular APs of the brainstem of the mouse brain slice. The brain slice is inserted and fixed in a chamber which contains artificial cerebrospinal fluid (ACSF) which is continuously oxygenated and kept humid to mimic a real brain environment and to keep the neurons alive for a few hours. The micropipette is gradually penetrated into the brainstem tissue by means of a microscope and its peripheral tools.

**Figure 20:**
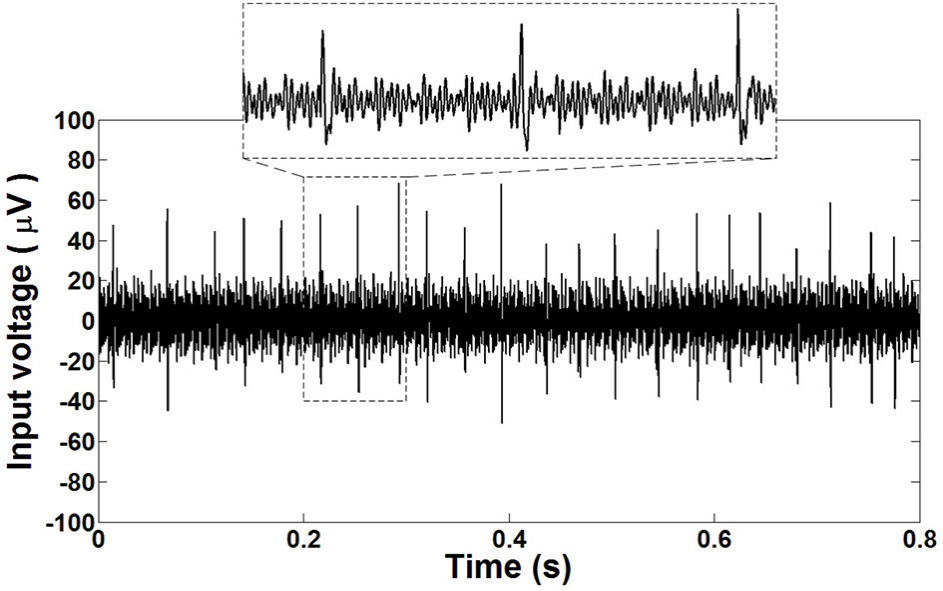
Recorded extracellular APs extracted from the brainstem of a mouse with the fabricated neural amplifier.

To complete the test setup, the AgCl electrode of the micropipette is connected to the non-inverting input of the prototype amplifier. The connection of the chamber, including the ACSF, is connected to the inverting port of the amplifier as a Vref. It should be noted that shielded wires are utilized to perform these connections. A commercial setup of a neural recording system containing an instrumentation amplifier (A-M systems, Inc.), rack mounted data acquisition equipment and a PC with a spike2 Windows-based software (version 5.19, Cambridge Electronic design) was utilized. The output of the proposed amplifier is connected to the commercial amplifier. The commercial amplifier is a band pass amplifier with a midband gain of 100 (V/V) and with low and high cutoff frequencies of 300 Hz and 5 kHz, respectively. Setting the low-cutoff frequency at 300 Hz allows us to eliminate the LFP and extract the extra cellular APs from the output signal. By using the commercial amplifier as the second stage amplifier, the total gain is achieved at 5,300 *V*/*V*. During the test procedure, the amplified signal is sampled with a frequency of 10 kS/s and digitized by the mentioned data acquisition equipment and transferred to the PC. Spike2 was used to observe the captured data in the PC. Figure 20 illustrates the recorded spontaneous extra cellular APs from the brainstem of the mouse with the proposed neural amplifier.

## 5. Conclusion

Scaling down technology introduces new challenges in neural amplifier design. One main challenge is the increased low-cutoff frequency (*f*_*L*_) of the AC-coupled amplifiers, assuming the same feedback capacitance value is used. The simplest solution is to increase the feedback capacitors. However, this comes at the cost of increased input capacitors for the same gain of the amplifier, which increases the silicon area and decreases the input impedance of the amplifier. Assuming a neural recording implant requires a large array of these amplifiers, the total consumption of the silicon area increases dramatically.

In this paper, we focus on this challenge, find its roots, and propose solutions to improve it. Scaling down the technology increases the leakage current of the differential pair of the OTA due to decreasing the gate oxide thickness (short channel effects). This is translated to decreasing the input resistance (*R*_*i*_) of OTA. We show, through simulations backed by an analytical analysis, that decreasing *R*_*i*_ is the fundamental reason for the increase in *f*_*L*_. Two different solutions are presented in this paper to increase *R*_*i*_: applying a cross-coupled positive feedback architecture and utilizing thick-oxide PMOS transistors in a differential pair of the OTA. The simulations confirm that both of the solutions decrease the *f*_*L*_. We designed and fabricated the latter solution in the 65 nm TSMC process. The experimental results show that the low-cutoff frequency decreases to 2 Hz with 208 fF feedback capacitor (*C*_*F*_). The neural amplifier is verified by *in vitro* experiment on mouse brainstem slices.

## Data Availability Statement

The original contributions presented in the study are included in the article/supplementary material, further inquiries can be directed to the corresponding author/s.

## Author Contributions

All authors listed have made a substantial, direct and intellectual contribution to the work, and approved it for publication.

## Conflict of Interest

The authors declare that the research was conducted in the absence of any commercial or financial relationships that could be construed as a potential conflict of interest.

## References

[B1] Abdelhalim K. Kokarovtseva L. Velazquez J. L. P. Genov R. (2013). 915-MHz FSK/OOK wireless neural recording SoC with 64 mixed-signal FIR filters. IEEE J. Solid State Circ. 48, 2478–2493. 10.1109/JSSC.2013.2272849

[B2] Bagheri A. Salam M. T. Velazquez J. L. P. Genov R. (2017). Low-frequency noise and offset rejection in DC-coupled neural amplifiers: a review and digitally-assisted design tutorial. IEEE Trans. Biomed. Circ. Syst. 11, 161–176. 10.1109/TBCAS.2016.2539518 27305685

[B3] Biederman W. Yeager D. J. Narevsky N. Koralek A. C. Carmena J. M. Alon E.. (2013). A fully-integrated, miniaturized (0.125 mm^2^) 10.5 μW wireless neural sensor. IEEE J. Solid State Circ. 48, 960–970. 10.1109/JSSC.2013.2238994

[B4] Biederman W. Yeager D. J. Narevsky N. Leverett J. Neely R. Carmena J. M.. (2015). A 4.78 *mm*^2^ fully-integrated neuromodulation soc combining 64 acquisition channels with digital compression and simultaneous dual stimulation. IEEE J. Solid State Circ. 50, 1038–1047. 10.1109/JSSC.2014.2384736

[B5] Cabrera C. Caballero R. Costa-Rauschert M. C. Rossi-Aicardi C. Oreggioni J. (2020). “Low-voltage low-noise high-CMRR biopotential integrated preamplifier,” in IEEE Transactions on Circuits and Systems I: Regular Papers, 1–10.

[B6] Cook M. J. O'Brien T. J. Berkovic S. F. Murphy M. Morokoff A. Fabinyi G.. (2013). Prediction of seizure likelihood with a long-term, implanted seizure advisory system in patients with drug-resistant epilepsy: a first-in-man study. Lancet Neurol. 12, 563–571. 10.1016/S1474-4422(13)70075-9 23642342

[B7] Denison T. Consoer K. Santa W. Avestruz A.-T. Cooley J. Kelly A. (2007). A 2 μW 100 nV$/\sqrt{Hz}$ chopper-stabilized instrumentation amplifier for chronic measurement of neural field potentials. IEEE J. Solid State Circ. 42, 2934–2945. 10.1109/JSSC.2007.908664

[B8] Enz C. C. Krummenacher F. Vittoz E. A. (1995). An analytical MOS transistor model valid in all regions of operation and dedicated to low-voltage and low-current applications. Analog Integr. Circ. Signal Process. 8, 83–114. 10.1007/978-1-4615-2283-6_7

[B9] Farouk T. Dessouky M. Elkhatib M. (2020). A fabrication of a low-power low-noise neural recording amplifier based on flipped voltage follower. Microelectron. J. 101:104817. 10.1016/j.mejo.2020.104817

[B10] Fifer M. Acharya S. Benz H. Mollazadeh M. Crone N. Thakor N. (2012). Toward electrocorticographic control of a dexterous upper limb prosthesis: building brain-machine interfaces. IEEE Pulse 3, 38–42. 10.1109/MPUL.2011.2175636 22344950 PMC3987748

[B11] Harrison R. R. Charles C. (2003). A Low-power low-noise CMOS amplifier for neural recording applications. IEEE J. Solid-State Circ. 38, 958–965. 10.1109/JSSC.2003.81197916285399

[B12] Hashemi Noshahr F. Nabavi M. Sawan M. (2020). Multi-channel neural recording implants: a review. Sensors 20:904. 10.3390/s20030904 32046233 PMC7038972

[B13] Hashemi Noshahr F. Sawan M. (2017). “A compact and low power bandpass amplifier for low bandwidth signal applications in 65-nm CMOS,” in IEEE International Symposium on Circuits and Systems (ISCAS), (Baltimore, MD: IEEE), 1–4.

[B14] Jomehei M. G. Sheikhaei S. (2019). A low-power low-noise CMOS bio-potential amplifier for multi-channel neural recording with active DC-rejection and current sharing. Microelectron. J. 83, 197–211. 10.1016/j.mejo.2018.11.021

[B15] Kassiri H. Abdelhalim K. Genov R. (2013). “Low-distortion super-GOhm subthreshold-MOS resistors for CMOS neural amplifiers,” in Biomedical Circuits and Systems Conference (BioCAS), (IEEE), 270–273.

[B16] Kim J. Ko H. (2019). Self-biased ultralow power current-reused neural amplifier with on-chip analog spike detections. IEEE Access 7, 109792–109803. 10.1109/ACCESS.2019.2933674

[B17] Lee B. Jia Y. Mirbozorgi S. A. Connolly M. Tong X. Zeng Z.. (2019). An inductively-powered wireless neural recording and stimulation system for freely-behaving animals. IEEE Trans. Biomed. Circ. Syst. 13, 413–424. 10.1109/TBCAS.2019.2891303 30624226 PMC6510586

[B18] Luan L. Robinson J. T. Aazhang B. Chi T. Yang K. Li X.. (2020). Recent advances in electrical neural interface engineering: minimal invasiveness, longevity, and scalability. Neuron 108, 302–321. 10.1016/j.neuron.2020.10.011 33120025 PMC7646678

[B19] Luo D. Zhang M. Wang Z. (2019). A low-noise chopper amplifier designed for multi-channel neural signal acquisition. IEEE J. Solid State Circ. 54, 2255–2265. 10.1109/JSSC.2019.2913101

[B20] Mollazadeh M. Murari K. Cauwenberghs G. Thakor N. V. (2009). Wireless micropower instrumentation for multimodal acquisition of electrical and chemical neural activity. IEEE Trans. Biomed. Circ. Syst. 3, 388–397. 10.1109/TBCAS.2009.2031877 23853286

[B21] Muller R. Gambini S. Rabaey J. M. (2012). A 0.013 mm^2^, 5 μW, DC-Coupled neural signal acquisition IC with 0.5 V supply. IEEE J. Solid State Circ. 47, 232–243. 10.1109/JSSC.2011.2163552

[B22] Musk E. (2019). An integrated brain-machine interface platform with thousands of channels. BioRXiv 703801. 10.2196/16194 PMC6914248 31642810

[B23] Najafi K. Wise K. D. (1986). An implantable multielectrode array with on-Chip signal processing. IEEE J. Solid State Circ. 21, 1035–1044. 10.1109/JSSC.1986.1052646

[B24] Ng K. A. Xu Y. P. (2012). “A compact, low input capacitance neural recording amplifier with C_in_/Gain of 20 fF.V/V,” in Biomedical Circuits and Systems Conference (BioCAS), (IEEE), 328–331. 10.1109/TBCAS.2013.2280066 24144666

[B25] Ng K. A. Xu Y. P. (2013). A compact, low input capacitance neural recording amplifier. IEEE Trans. Biomed. Circ. Syst. 7, 610–620. 10.1109/TBCAS.2013.2280066 24144666

[B26] Ng K. A. Xu Y. P. (2016). A low-power, high cmrr neural amplifier system employing cmos inverter-based otas with cmfb through supply rails. IEEE J. Solid State Circ. 51, 724–737. 10.1109/JSSC.2015.2512935

[B27] Razavi B. (2005). Design of Analog CMOS Integrated Circuits. New York, NY: McGraw-Hill Education.

[B28] Rezaee-Dehsorkh H. Ravanshad N. Lotfi R. Mafinezhad K. Sodagar A. M. (2011). Analysis and design of tunable amplifiers for implantable neural recording applications. IEEE Trans. Emerg. Sel. Top. Circ. Syst. 1, 546–556. 10.1109/JETCAS.2011.2174492

[B29] Samiei A. Hashemi H. (2019). A chopper stabilized, current feedback, neural recording amplifier. IEEE Solid State Circ. Lett. 2, 17–20. 10.1109/LSSC.2019.2916754 33748689 PMC7970809

[B30] Schwartz A. B. Cui X. T. Weber D. J. Moran D. W. (2006). Brain-controlled interfaces: movement restoration with neural prosthetics. Neuron 52, 205–220. 10.1016/j.neuron.2006.09.019 17015237

[B31] Shoaran M. Kamal M. H. Pollo C. Vandergheynst P. Schmid A. (2014). Compact low-power cortical recording architecture for compressive multichannel data acquisition. IEEE Trans. Biomed. Circ. Syst. 8, 857–870. 10.1109/TBCAS.2014.2304582 24723633

[B32] Song S. Rooijakkers M. Harpe P. Rabotti C. Mischi M. Van Roermund A.. (2013). “A 430nw 64nv/vhz current-reuse telescopic amplifier for neural recording applications,” in 2013 IEEE Biomedical Circuits and Systems Conference (BioCAS), (Rotterdam: IEEE), 322–325.

[B33] Stevenson I. H. Kording K. P. (2011). How advances in neural recording affect data analysis. Nat. Neurosci. 14:139. 10.1038/nn.2731 21270781 PMC3410539

[B34] Sun F. Morrell M. Wharen R. J. (2008). Responsive cortical stimulation for the treatment of epilepsy. Neurotherapeutics 5, 68–74. 10.1016/j.nurt.2007.10.069 18164485 PMC5084128

[B35] Van Rijn A. C. M. Peper A. Grimbergen C. A. (1991). High-quality recording of bioelectric events. Med. Biol. Eng. Comput. 29, 433–440. 10.1007/BF02441666 1787761

[B36] Verma N. Shoeb A. Bohorquez J. Dawson J. Guttag J. Chandrakasan A. P. (2010). A micro-power EEG acquisition SoC with integrated feature extraction processor for a chronic seizure detection system. IEEE J. Solid State Circ. 45, 804–816. 10.1109/JSSC.2010.2042245

[B37] Xiao Z. Tang C.-M. Dougherty C. M. Bashirullah R. (2010). “A 20μw neural recording tag with supply-current-modulated afe in 0.13 μm cmos,” in 2010 IEEE International Solid-State Circuits Conference-(ISSCC) (IEEE), 122–123.

[B38] Xu J. Yazicioglu R. F. Grundlehner B. Harpe P. Makinwa K. A. Van Hoof C. (2011). A 160 μW 8-channel active electrode system for EEG monitoring. IEEE Trans. Biomed. Circ. Syst. 5, 555–567. 10.1109/TBCAS.2011.2170985 23852553

[B39] Yazicioglu R. F. Kim S. Torfs T. Kim H. Van Hoof C. (2011). A 30 μW analog signal processor ASIC for portable biopotential signal monitoring. IEEE J. Solid State Circ. 46, 209–223. 10.1109/JSSC.2010.2085930

[B40] Yazicioglu R. F. Merken P. Puers R. Van Hoof C. (2008). A 200 μW eight-channel EEG acquisition ASIC for ambulatory EEG systems. IEEE J. Solid State Circ. 43, 3025–3038. 10.1109/JSSC.2008.2006462

[B41] Zou X. Xu X. Yao L. Lian Y. (2009). A 1-v 450-nw fully integrated programmable biomedical sensor interface chip. IEEE J. Solid State Circ. 44, 1067–1077. 10.1109/JSSC.2009.2014707

